# Light-Driven Topological and Magnetic Phase Transitions in Thin Layer Antiferromagnets

**DOI:** 10.1021/acs.jpclett.2c00070

**Published:** 2022-05-04

**Authors:** Martin Rodriguez-Vega, Ze-Xun Lin, Aritz Leonardo, Arthur Ernst, Maia G. Vergniory, Gregory A. Fiete

**Affiliations:** † Theoretical Division, Los Alamos National Laboratory, Los Alamos, New Mexico 87545, United States; ‡ Department of Physics, The University of Texas at Austin, Austin, Texas 78712, United States; § Department of Physics, Northeastern University, Boston, Massachusetts 02115, United States; ∥ Donostia International Physics Center, Paseo Manuel de Lardizabal 4, 20018 San Sebastian, Spain; ⊥ EHU Quantum Center, University of the Basque Country UPV/EHU, 48940 Leioa, Spain; # Institut für Theoretische Physik, Johannes Kepler Universität, A 4040 Linz, Austria; @ Max-Planck-Institut für Mikrostrukturphysik, Weinberg 2, D-06120 Halle, Germany; ∇ Max Planck Institute for Chemical Physics of Solids, Dresden D-01187, Germany; ● Department of Physics, Massachusetts Institute of Technology, Cambridge, Massachusetts 02139, United States

## Abstract

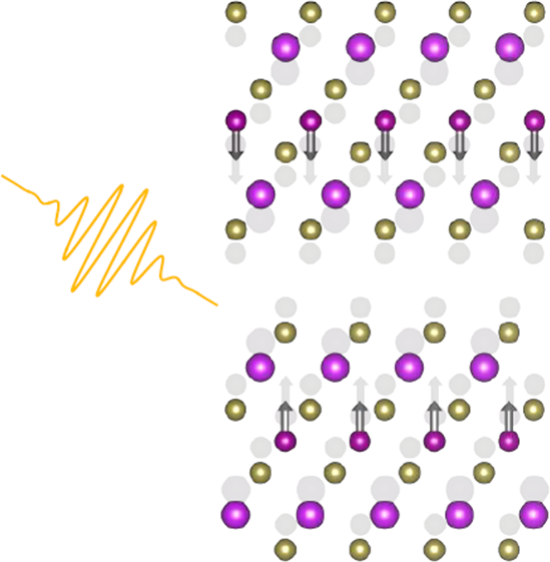

We theoretically study the effect of low-frequency light pulses in resonance with phonons in the topological and magnetically ordered two-septuple layer (2-SL) MnBi_2_Te_4_ (MBT) and MnSb_2_Te_4_ (MST). These materials share symmetry properties and an antiferromagnetic ground state in pristine form but present different magnetic exchange interactions. In both materials, shear and breathing Raman phonons can be excited via nonlinear interactions with photoexcited infrared phonons using intense laser pulses that can be attained in the current experimental setups. The light-induced transient lattice distortions lead to a change in the sign of the effective interlayer exchange interaction and magnetic order accompanied by a topological band transition. Furthermore, we show that moderate antisite disorder, typically present in MBT and MST samples, can facilitate such an effect. Therefore, our work establishes 2-SL MBT and MST as candidate platforms for achieving non-equilibrium magneto-topological phase transitions.

Antiferromagnetic topological insulators (ATIs) can host exotic phases of matter such as the quantum anomalous Hall (QAH) effect and axion insulators.^1^ The search for these topological phases motivated the addition of magnetic dopants in topological insulators, which led to the observation of a QAH effect and candidates for axion insulators at very low temperatures.^2−4^ However, intrinsic ATIs promise to manifest these phases at higher temperatures, which are desirable for applications. Indeed, the recent predictions, synthesis, and exfoliation of the van der Waals materials MnBi_2_Te_4_, MnBi_2*n*_Te_3*n*+1_, and MnSb_2_Te_4_^5−12^ allowed the detection of QAH states in odd septuple layers (SLs) and axion states in even SLs^13−17^ and the observation of an electric field-induced layer Hall effect in six SL samples.^18^

The intertwined nature of the magnetic and topological order in ATIs offers the possibility of exploring topological transitions induced by changes in the magnetic order and vice versa. For example, recent experiments suggest that increasing the distance between the magnetic planes in the MnBi_2*n*_Te_3*n*+1_ family leads to ferromagnetic order.^8^ On the contrary, decreasing the distance in MnBi_2_Te_4_ single crystals via hydrostatic pressure leads to the suppression of the AFM order.^19,20^ In contrast, in CrI_3_, a low-dimensional magnetic system with trivial topology, hydrostatic pressure induces an antiferromagnet (AFM) to ferromagnet (FM) transition.^21^ However, a suitable mechanism for modifying the magnetic order in ATIs without applied external magnetic fields or superlattices remains elusive.

To this end, non-equilibrium approaches provide a possible pathway for achieving magneto-topological transitions in ATIs.^22−25^ Most notably, nonlinear phononics,^22,26,27^ a transient and controlled lattice distortion induced by photoexcited phonons, has been successfully used to transiently enhance superconductivity,^28−30^ manipulate and induce ferroelectric states,^31,32^ and induce dynamical ferrimagnetic transitions.^33^ More recently, Stupakiewicz et al. induced switching of magnetization in yttrium iron garnet (YIG) thin films by pumping of phonon modes.^34^ More generally, light has been shown to induce metastable charge-density-wave states^35^ and incite transitions into hidden phases.^36^ This experimental evidence motivates the use of non-equilibrium approaches to manipulate magneto-topological order in ATIs. In this work, we show theoretically that an AFM to FM magnetic transition accompanied by a topological transition can be induced in 2-SL MXT (X = Bi or Sb) samples with intense, experimentally accessible terahertz laser pulses in resonance with the phonons. Interestingly, the moderate antisite disorder typically present in these materials reduces the laser intensity threshold to induce the transition.

In MXT materials, the constituent SLs (see Figure 1a) are held together via van der Waals forces, which allows exfoliation in thin samples.^37,38^ We will focus on systems with two SLs, because they correspond to the minimal system that can accommodate interlayer AFM order. Within each layer, the magnetic moments are aligned ferromagnetically, but opposite layers possess opposite magnetic moment directions. For 2-SL MBT, the critical temperature is approximately 20 K.^39^ For bulk MST, a critical temperature of 19 K has been reported.^40^ However, depending on the synthesis conditions, bulk MST can possess a ferromagnetic ground state.^41,42^

**Figure 1:**
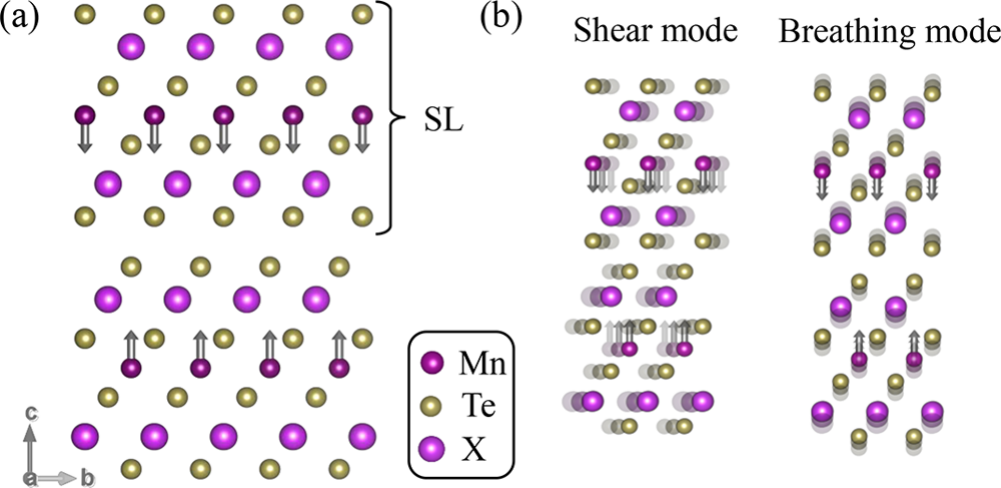
(a) 2-SL MXT lattice structure and magnetic order (moments shown as gray arrows). Bi and Sb (X) atoms are colored pink, Te atoms yellow, and Mn atoms purple. (b) Low-frequency shear and breathing modes characteristic of few-layer materials. The breathing mode preserves all of the crystal symmetries.

2-SL MBT and 2-SL MST present space group *P*3̅*m*1 (No. 164) with point group *D*_3*d*_ in their paramagnetic phase. The unit cell contains *N* = 14 atoms with Te atoms located in Wyckoff positions 2*d* (^1^/_3_, ^2^/_3_, *z*) and 2*c* (0, 0, *z*), Mn atoms at position 2*d*, and X = Bi and Sb atoms at positions 2*c* and 2*d*. The lattice vibration representation is given by the equation Γ_vib_ = 7*A*_1*g*_ ⊕ 7*A*_2*u*_ ⊕ 7*E*_*g*_ ⊕ 7*E*_*u*_, which corresponds to seven nondegenerate (*A*_1*g*_) and seven double-degenerate (*E*_*g*_) Raman modes, with equal numbers of their infrared counterparts, including the three acoustic modes (*E*_*u*_ ⊕ *A*_2*u*_). The character table for *D*_3*d*_ is shown in the Supporting Information.

Employing group theory and projection operators, we derive the set of real-space displacements that bring the dynamical matrix into block-diagonal form, according to their irreducible representations (see the methods in the Supporting Information for details). We find that the shear mode where one SL shifts in the [100] direction and the opposite SL in the [1̅00] direction belongs to the *E*_*g*_ irrep. Its partner corresponds to an orthogonal in-plane displacement. The breathing mode consists of the SLs moving away from and toward each other in the direction normal to the plane ([001] and [001̅], respectively) and belongs to the *A*_1*g*_ representation. Figure 1b shows representations of these modes. For a detailed group theory study of few-SL MBT, see ref (43).

Now that we have established that the shear and breathing modes are allowed by symmetry and determined their irreps, we calculate the phonon frequencies at the Γ point. We considered paramagnetic, FM, and AFM configurations without spin–orbit coupling and found only negligible differences among the corresponding phonon frequencies. The results for both 2-SL MBT and MST are summarized in Figure 2. Panels a and b show the Γ point phonon frequencies with their corresponding irreducible representation indicated by the shape of the marker. In both materials, the shear and breathing modes present the smallest frequency among the optical modes (indicated by downward gray arrows), and their frequency is smaller by a factor of 2 compared with that of the next optical phonon.

**Figure 2:**
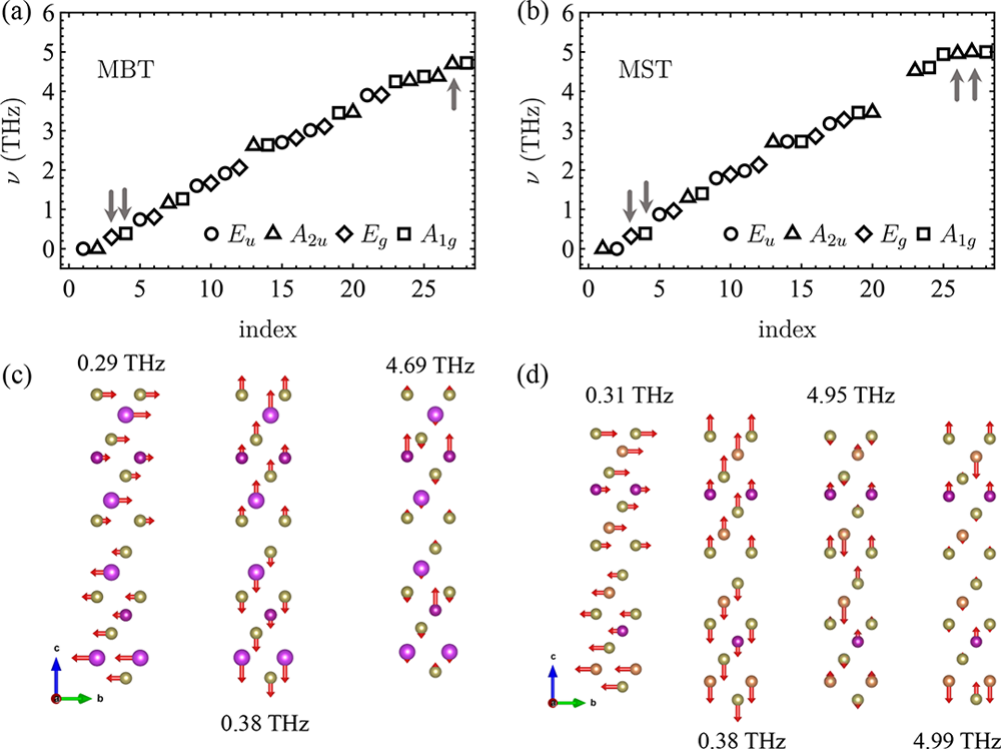
Phonon frequencies for (a) 2-SL MBT and (b) 2-SL MST obtained with first-principles calculations. The gray arrows indicate the phonons illustrated below. (c and d) Real-space lattice displacements with their corresponding frequencies. Red arrows indicate the displacements.

Having characterized the properties of the phonons in the harmonic regime, we next consider the symmetry aspects of their nonlinear interactions and their laser excitation. A laser pulse incident onto a sample can couple directly with infrared (IR) modes, depending on the laser frequency and electric field direction. In turn, such an IR mode can couple nonlinearly with some Raman modes, provided their irreps satisfy the condition [Γ_IR_ ⊗ Γ_IR_] ⊗ Γ_R_ ⊃ *A*_1*g*_.^27^ This mechanism is termed nonlinear phononics^26,27,44^ and has allowed experimental^22,28−30,32,44,45^ and theoretical manipulations of correlated states of matter.^46−49^ For the 2-SL MXT’s point group, driving a *A*_2*u*_ mode can rectify totally symmetric modes, such as the breathing modes, because *A*_2*u*_ ⊗ *A*_2*u*_ = *A*_1*g*_. Thus, the shear modes (*E*_*g*_ irrep) are not affected. On the contrary, driving an *E*_*u*_ mode allows coupling with the low-frequency shear modes in conjunction with the breathing mode, because *E*_*u*_ ⊗ *E*_*u*_ = *A*_1*g*_ ⊕ *A*_2*g*_ ⊕ *E*_*g*_.

Once an IR mode has been driven with a sufficiently strong laser pulse, coupling to all Raman modes with compatible irreps is allowed by symmetry. However, in our case, because the solution of the dynamical equations scales with the inverse square of the Raman frequency (∼Ω_R_^–2^), we can simplify the calculation and restrict the nonlinear interactions to only the low-frequency shear and breathing modes.^27,48^ We now consider a laser pulse optimized to couple with the highest-frequency IR modes, with irrep *A*_2*u*_. This mode presents the strongest coupling with the laser as shown by the largest Born effective charge Z*^50,51^ (see the Supporting Information). In this case, the nonlinear potential for 2-SL MBT takes the form
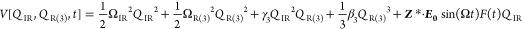
1where γ_3_ and β_3_ are nonlinear coefficients determined from DFT calculations (for the procedure and numerical values, see the Supporting Information), *E*_0_ is the electric field amplitude with the Gaussian profile *F*(*t*) = exp[−*t*^2^/(2τ^2^)], and Ω is the laser frequency, which we choose in resonance with the IR mode Ω = Ω_IR_ = 4.69 THz. Note that the driven *A*_2*u*_ nonlinear potential is much simpler than that for driven *E*_*u*_ modes. This is because the *A*_2*u*_ phonons do not couple to *E*_*g*_ modes up to cubic-order interactions.

For 2-SL MST, there are two IR modes with *A*_2*u*_ irreps, similar Born effective charges, and similar frequencies. Therefore, we need to consider the simultaneous excitation of the two *A*_2*u*_ IR modes, which leads to the potential
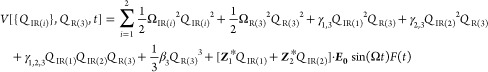
2

For 2-SL MST, we consider the laser frequency Ω = [Ω_IR(1)_ + Ω_IR(2)_]/2 THz. The phonon dynamics are determined by the equations of motion ∂_*t*_^2^*Q*_R_ = −∂_*Q*_R__*V*[*Q*_IR(*i*)_, *Q*_R_] and ∂_*t*_^2^*Q*_IR(*i*)_ = −∂_*Q*_IR(*i*)__*V*[*Q*_IR(*i*)_, *Q*_R_], where *i* runs over the driven IR modes. We solve the differential equations numerically. In this work, we do not consider the phonon lifetime. Recent Raman measurements have shown that the lifetime of the breathing mode is approximately 13.3 ps,^52^ which is sufficiently long for the electronic degrees of freedom to respond.

The phonon dynamics for a general laser intensity and pulse duration can be obtained by solving the equations of motion numerically. In Figure 3a, we show a sketch of a laser-irradiated 2-SL MBT sample. The incoming light with frequency Ω = Ω_IR_ = 4.69 THz couples directly to the corresponding resonant IR mode. As we show in Figure 3b, this mode oscillates around its equilibrium position. Anharmonic coupling induces dynamics in the Raman breathing mode, even though it does not couple directly to the laser. The nonlinear nature of the interaction (γ_3_*Q*_R_*Q*_IR_^2^) leads to oscillations about a position shifted with respect to the equilibrium position. Figure 3c shows such oscillations for a laser with peak electric field *E*_0_ = 0.6 and pulse duration τ = 0.6 ps. Similar responses were obtained in 2-SL MST, where the main difference is the presence of two *A*_2*u*_ IR modes, instead of one. Notice that with light, we can obtain only ⟨*Q*_R(3)_⟩ ≥ 0, which corresponds to an effective increase in the Mn–Mn layer separation. This is a consequence of the sign of the nonlinear coefficients (γ_3_ for MBT and γ_1,3_ and γ_2,3_ for MST), which is intrinsic for the materials.

**Figure 3:**
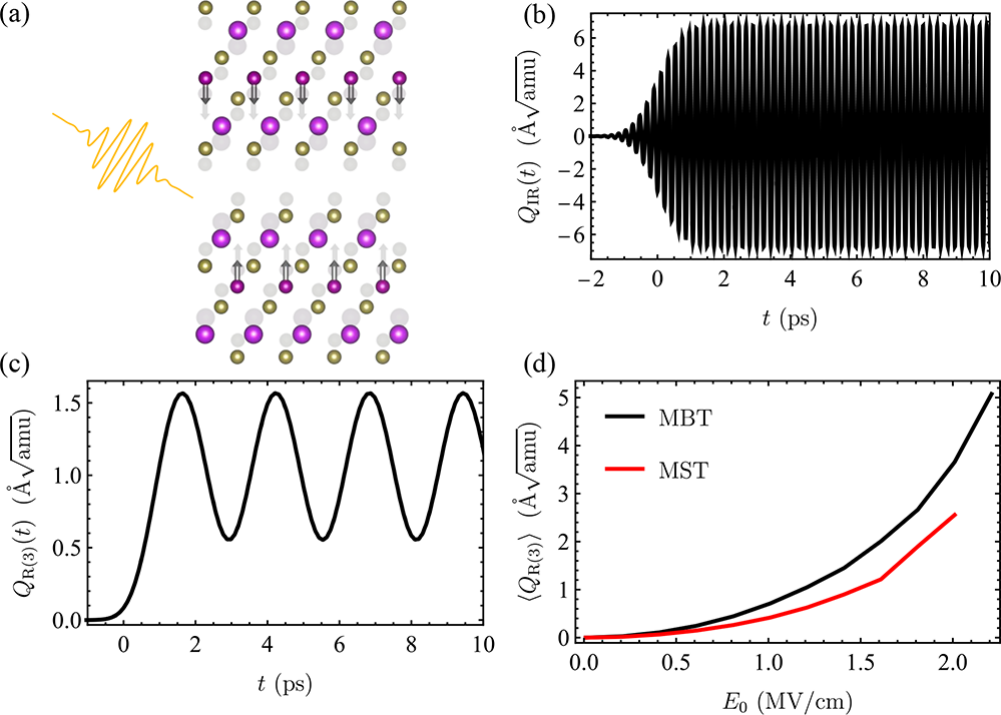
(a) Sketch of a light-induced lattice distortion. (b) Time dependence of the infrared phonon mode directly excited by the incident laser pulse in 2-SL MBT. (c) Nonlinearly excited breathing mode, which oscillates about a new shifted position. The laser parameters used in panels b and c are τ = 0.6 ps and *E*_0_ = 0.6 MV/cm. (d) Average displacement of the nonlinearly photoexcited breathing mode *Q*_R(3)_ for MBT (black) and MST (red) for τ = 0.3 ps and laser frequency Ω = Ω_IR(1)_ for 2-SL MBT and Ω = [Ω_IR(1)_ + Ω_IR(2)_]/2 for 2-SL MST.

The complementary process of bringing the Mn planes closer to each other could be achieved by applying uniaxial pressure. Theoretically, ref (53) predicts that bulk MBT undergoes a topological quantum phase transition under 2.12% compressive strain.

In Figure 3d, we plot the time average of the shear modes as a function of *E*_0_ for τ = 3 ps. Experimentally, fields of ≤100 MV cm^–1^ have been reported in the range of 15–50 THz,^54,55^ but limitations are imposed by the amplitude of the corresponding lattice distortion. For 2-SL MBT (2-SL MST), ⟨*Q*_R(3)_⟩ = 5 Å/ corresponds to a 1.68% (1.88%) increase in the Mn–Mn plane interlayer distance. For 2-SL MST, the dynamical equations become unstable for *E*_0_ ≳ 2 MV/cm. However, the range of stability is large enough to obtain a magnetic transition.

Inelastic neutron scattering measurements^56^ suggest that the magnetic order in bulk MBT is described by the local-moment Hamiltonian (*S* = ^5^/_2_) , where the intralayer Hamiltonian can be written as  with exchange interaction *J*_*ij*_ (up to fourth-neighbor interactions are needed to fit the data correctly with *SJ*_1_ = 0.3 meV, *SJ*_2_ = −0.083 meV, and *SJ*_4_ = 0.023 meV), and SD = 0.12 meV is a single-ion anisotropy. Thus, the effective intralayer coupling is positive and leads to the ferromagnetic order in each Mn layer. The interlayer Hamiltonian is given by , where experiments suggest a nearest-neighbor AFM interlayer interaction *SJ*_c_ = −0.055 meV.^56^ We obtain the spin Hamiltonian from first-principles calculations, employing a Green’s function approach and the magnetic force theorem.^57,58^ The calculations were performed using a GGA+U approximation, which describes adequately localized Mn 3d states with *U*_eff_ = *U* – *J* = 5.3 eV.^5,41^ For the interlayer interactions, the Hamiltonian takes the more general form , where longer-range interactions are relevant. In pristine MXT compounds, the interlayer coupling governs the antiferromagnetic order in the ground state, which is mainly mediated by a long-range double-exchange interaction via Te ions.^5,41^ However, natural lattice defects such as antisite Mn–Bi or Mn–Sb disorder or Mn excess in Bi (Sb) layers can lead to ferromagnetic order in these systems.^41,59^

We now study the effect of laser-induced transient lattice distortions on the magnetic order. Under a time-dependent lattice deformation, small compared with the equilibrium interatomic distances, the spin exchange interaction can be approximated as^60^
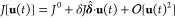
3where **u**(*t*) is the real-space lattice displacement, *J*^0^ is the equilibrium interaction, and *δJ* is the coupling constant between the phonon and the spins. The connection with phonon amplitude *Q* is given by , where *m*_κ_ is the mass of atom κ and **e**_κ_ is the normalized dynamical matrix eigenvectors.

Next, we define the effective spin interaction employing Floquet theory. The exchange interactions set the relevant energy scale, with ≲1 meV. Because the infrared phonon frequency (Ω_IR_ ≈ 4.95 and 4.69 THz) used is larger than the exchange energy, we can define an effective time-averaged exchange interaction *J*^eff^ = *J*^0^ + *δJ***δ̂**·⟨**u**_R_⟩, where ⟨···⟩ indicates the time average. Thus, when the phonons oscillate about their equilibrium positions (harmonic phonons), such that ⟨**u**⟩ = 0, the exchange interactions are not modified in the picture discussed here. The non-zero average shift, however, can renormalize the interactions leading to different magnetic configurations compared with the equilibrium counterparts.

We compute the light-induced effective exchange interactions as a function of phonon amplitude *Q*_R(3)_. Our results are summarized in Figure 4. We plot the average interlayer exchange interaction  as a function of *Q*_R(3)_. We used a supercell, which consists of seven SLs of MBT (MST) and three SLs of vacuum simulated by empty spheres. *J̅*_eff_ represents an average exchange interaction, and  is the number of interacting magnetic moments taken for the average. For pristine 2-SL MBT (2-SL MST), we find a sign change in the interlayer exchange interaction at *Q*_R(3)_ ≈ 2.4 Å [*Q*_R(3)_ ≈ 0.7 Å]. These phonon amplitudes can be obtained with a laser pulse with an *E*_0_ of ≈1.7 MV/cm and a τ of 0.3 ps (*E*_0_ ≈ 1.5 MV/cm, and τ = 0.3 ps), as we show in Figure 3. Generally, increasing the vertical distance between the Mn magnetic moments weakens the antiferromagnetic coupling and favors ferromagnetic order in these systems. The time scale for the spin reorientation following the sign change in *J̅*_eff_ depends on parameters such as the Gilbert damping factor,^61^ the exact spin anisotropy for 2-SL MBT and MST, and the laser-induced *J̅*_eff_ but is within the limits of the effect we predict to occur.

**Figure 4:**
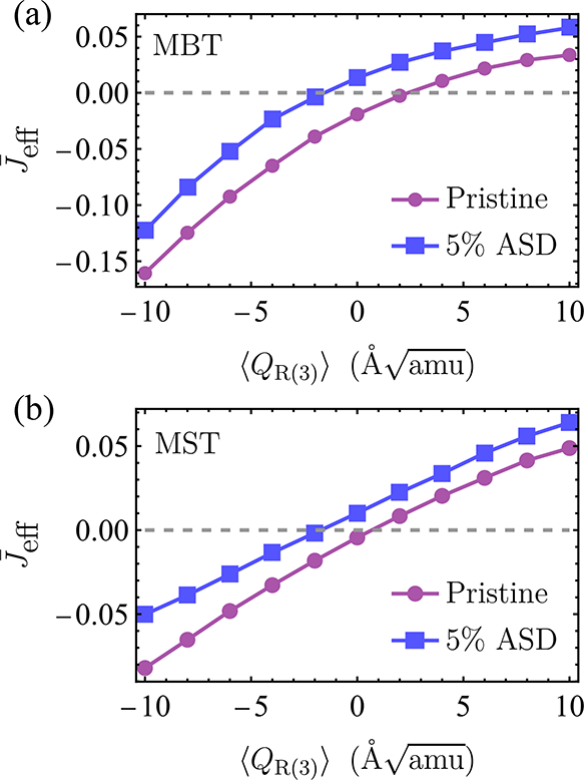
Effective averaged interlayer exchange interaction as a function of average breathing mode ⟨*Q*_R(3)_⟩ for (a) 2-SL MBT and (b) 2-SL MST. The purple circles correspond to pristine samples, while squares correspond to 5% antisite disorder (ASD).

Because MXT samples are prone to antisite disorder,^41,59,62−65^ with disorder percentages depending on the sample fabrication process, we also discuss the role of disorder in the light-induced magnetic transition. Depending on the concentration percentage, Mn–Sb antisite disorder can tune the interlayer magnetic interaction into ferromagnetic states.^63^ Here, we study theoretically the role of antisite disorder in the light-induced magnetic transition discussed previously.

First, we will assume that the antisite disorder has a negligible effect on the phonon frequencies. This assumption is supported by recent Raman measurements in 2-SL MBT samples with inherent antisite disorder, because the measured phonon frequencies are in agreement with density functional calculations for pristine samples.^52^

Next, we introduce disorder into our calculations for the exchange interactions. The antisite disorder is assumed to be an interchange of Mn with Bi(Sb) elements between the Mn layer and Bi(Sb) layers. This is consistent with recent experiments.^41^ Antisite disorder effects were found to have a quantitatively important effect on the exchange interaction in these materials. Disorder effects are treated using a coherent potential approximation (CPA) as it is implemented within multiple scattering theory.^66^ We show our results in Figure 4, where we consider 5% antisite disorder, which is a realistic concentration in most of the known MXT samples.^5,41,63^ In general, antisite disorder favors a ferromagnetic interlayer coupling. The main reason for this is that Mn moments in Bi(Sb) layers favor a long-range ferromagnetic coupling between the septuple layers.^67^ Also, the reduction of magnetic moments in Mn layers diminishes the extent of antiferromagnetic coupling. At zero displacement, a finite amount of disorder can weaken the effective exchange interaction, leading to weaker electric fields that are necessary to drive the transition. In Figure 4, the concentration we consider leads to a disorder-induced ferromagnetic ground state.

We established theoretically the possibility of tuning the interlayer magnetic order from antiferromagnetic to ferromagnetic in 2-SL MXT samples using light in resonance with the phonons. Now we demonstrate that a topological transition accompanies such a light-induced magnetic transition.

The topology in MBT is rich. In bulk MBT, the magnetic structure is invariant with respect to time reversal and half-lattice translation symmetries. This leads to a  topological classification, with .^5^ In the thin-film limit, the topology depends in the number of SLs.^68^ For example, 1-SL MBT is predicted to be a FM trivial insulator, with Chern number *C* = 0. 2-SL, 4-SL, and 6-SL MBT present a zero plateau QAH, with *C* = 0 in the AFM phase and |*C*| = 1 in the FM phase. Odd layer (3-, 5-, and 7-SL) MBT is predicted to be in a |*C*| = 1 QAH insulating state. Experimentally, the QAH state has been observed in 5-SL MBT at 1.4 K^37^ and a zero Hall plateau, characteristic of an axion insulating state, in 6-SL MBT.^38^

We study the topology of 2-SL MBT as a function of the lattice displacements by examining the electronic band structure and the projection of the p X = Bi, Sb, and Te states. The band inversion serves as an indicator of the topological nature of the material within topological band theory.^69^ Our results are summarized in Figure 5. In the equilibrium configuration (left panels with *Q* = 0) with FM order, both 2-SL MBT and 2-SL MST exhibit the expected band inversion.^5,41^ For the out-of-equilibrium distorted structures (right panels), FM order is preferred as we showed before. We find that the band inversion is present, which indicates the topological nature of the new laser-induced structures.

**Figure 5:**
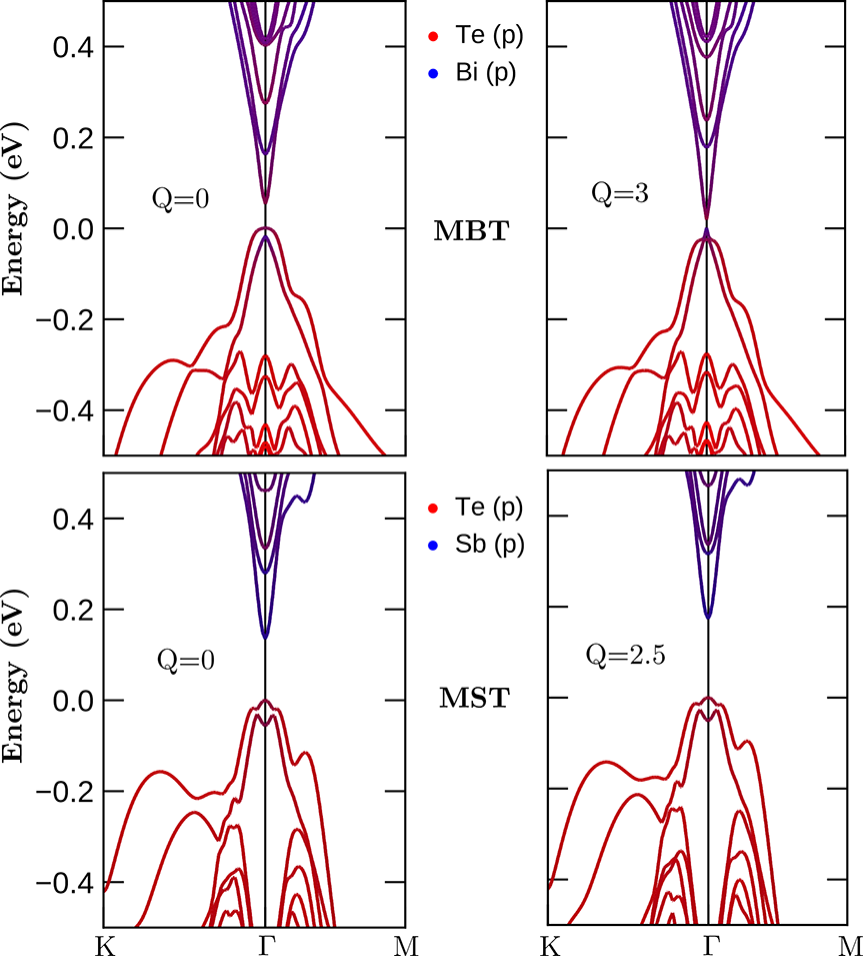
Band structure with projected p states for 2SL-MXT in the FM state. In all cases [FM static (*Q* = 0) and FM out of equilibrium (*Q* ≠ 0)], we find that the bands are inverted.

This work studied the effect of terahertz light pulses in resonance with infrared phonons in the magnetic and topological order of 2-SL MXT samples theoretically. We found that moderate laser intensities, which can be attained in current experimental setups, can induce nonlinear dynamics in the Raman breathing mode. The time average of these dynamics leads to effective lattice distortions that separate the SLs, effectively increasing the distance between magnetic atom planes. Using first-principles methods, we found that the new non-equilibrium lattice configuration can favor ferromagnetic order. Furthermore, the transition between antiferromagnetic and magnetic order can be tuned via antisite disorder. We showed that the magnetic change is accompanied by a topological transition, as diagnosed by a band inversion as a function of phonon amplitude. Thus, our theoretical work demonstrates the possibility of achieving a sought-after magnetic topological transition in 2-SL MXT samples experimentally. Such a transition in both 2-SL MBT and MST establishes a broader trend in materials, which could be applied to other van der Waals magnetic topological materials.

## Supporting Information Available

Additional details about the group theory phonon symmetry analysis, character table for the crystal point group, phonon first-principles calculations, and a discussion of the single-particle excitation spectrum (PDF)
Transparent Peer Review report available (PDF)

## References

[ref1] Mong R. S. K.; Essin A. M.; Moore J. E. Antiferromagnetic topological insulators. Phys. Rev. B 2010, 81, 245209 10.1103/PhysRevB.81.245209.

[ref2] Chang C.-Z.; et al. Experimental Observation of the Quantum Anomalous Hall Effect in a Magnetic Topological Insulator. Science 2013, 340, 167 10.1126/science.1234414. 23493424

[ref3] Mogi M.; Kawamura M.; Yoshimi R.; Tsukazaki A.; Kozuka Y.; Shirakawa N.; Takahashi K. S.; Kawasaki M.; Tokura Y. A magnetic heterostructure of topological insulators as a candidate for an axion insulator. Nat. Mater. 2017, 16, 516–521. 10.1038/nmat4855. 28191899

[ref4] Gooth J.; Bradlyn B.; Honnali S.; Schindler C.; Kumar N.; Noky J.; Qi Y.; Shekhar C.; Sun Y.; Wang Z.; Bernevig B. A.; Felser C. Axionic charge-density wave in the Weyl semimetal (TaSe4)2I. Nature 2019, 575, 315–319. 10.1038/s41586-019-1630-4. 31590178

[ref5] Otrokov M. M.; et al. Prediction and observation of an antiferromagnetic topological insulator. Nature 2019, 576, 416–422. 10.1038/s41586-019-1840-9. 31853084

[ref6] Gong Y.; et al. Experimental Realization of an Intrinsic Magnetic Topological Insulator. Chin. Phys. Lett. 2019, 36, 076801 10.1088/0256-307X/36/7/076801.

[ref7] Hu C.; et al. A van der Waals antiferromagnetic topological insulator with weak interlayer magnetic coupling. Nat. Commun. 2020, 11, 97 10.1038/s41467-019-13814-x. 31911588 PMC6946652

[ref8] Hu C.; et al. Realization of an intrinsic ferromagnetic topological state in MnBi_8_Te_13_. Sci. Adv. 2020, 6, eaba4275 10.1126/sciadv.aba4275. 32743072 PMC7375807

[ref9] Jahangirli Z. A.; Alizade E. H.; Aliev Z. S.; Otrokov M. M.; Ismayilova N. A.; Mammadov S. N.; Amiraslanov I. R.; Mamedov N. T.; Orudjev G. S.; Babanly M. B.; Shikin A. M.; Chulkov E. V. Electronic structure and dielectric function of Mn-Bi-Te layered compounds. J. Vac. Sci. Technol. B 2019, 37, 062910 10.1116/1.5122702.

[ref10] Aliev Z. S.; Amiraslanov I. R.; Nasonova D. I.; Shevelkov A. V.; Abdullayev N. A.; Jahangirli Z. A.; Orujlu E. N.; Otrokov M. M.; Mamedov N. T.; Babanly M. B.; Chulkov E. V. Novel ternary layered manganese bismuth tellurides of the MnTe-Bi_2_Te_3_ system: Synthesis and crystal structure. J. Alloys Compd. 2019, 789, 443–450. 10.1016/j.jallcom.2019.03.030.

[ref11] Ge W.; Sass P. M.; Yan J.; Lee S. H.; Mao Z.; Wu W. Direct evidence of ferromagnetism in MnSb_2_Te_4_. Phys. Rev. B 2021, 103, 134403 10.1103/PhysRevB.103.134403.

[ref12] Xu Y.; Elcoro L.; Song Z.-D.; Wieder B. J.; Vergniory M. G.; Regnault N.; Chen Y.; Felser C.; Bernevig B. A. High-throughput calculations of magnetic topological materials. Nature 2020, 586, 702–707. 10.1038/s41586-020-2837-0. 33116291

[ref13] Liu C.; Wang Y.; Li H.; Wu Y.; Li Y.; Li J.; He K.; Xu Y.; Zhang J.; Wang Y. Robust axion insulator and Chern insulator phases in a two-dimensional antiferromagnetic topological insulator. Nat. Mater. 2020, 19, 522 10.1038/s41563-019-0573-3. 31907415

[ref14] Deng Y.; Yu Y.; Shi M. Z.; Guo Z.; Xu Z.; Wang J.; Chen X. H.; Zhang Y. Quantum anomalous Hall effect in intrinsic magnetic topological insulator MnBi_2_Te_4_. Science 2020, 367, 895–900. 10.1126/science.aax8156. 31974160

[ref15] Ovchinnikov D.; et al. Intertwined Topological and Magnetic Orders in Atomically Thin Chern Insulator MnBi_2_Te_4_. Nano Lett. 2021, 21, 2544–2550. 10.1021/acs.nanolett.0c05117. 33710884

[ref16] Ge J.; Liu Y.; Li J.; Li H.; Luo T.; Wu Y.; Xu Y.; Wang J. High-Chern-number and high-temperature quantum Hall effect without Landau levels. Natl. Sci. Rev. 2020, 7, 1280–1287. 10.1093/nsr/nwaa089. 34692156 PMC8289033

[ref17] Lüpke F.; Pham A. D.; Zhao Y.-F.; Zhou L.-J.; Lu W.; Briggs E.; Bernholc J.; Kolmer M.; Teeter J.; Ko W.; Chang C.-Z.; Ganesh P.; Li A.-P. Local manifestations of thickness dependent topology and axion edge state in topological magnet MnBi_2_Te_4_. Phys. Rev. B 2022, 105, 035423 10.1103/PhysRevB.105.035423.

[ref18] Gao A.; et al. Layer Hall effect in a 2D topological axion antiferromagnet. Nature 2021, 595, 521–525. 10.1038/s41586-021-03679-w. 34290425

[ref19] Chen K. Y.; Wang B. S.; Yan J.-Q.; Parker D. S.; Zhou J.-S.; Uwatoko Y.; Cheng J.-G. Suppression of the antiferromagnetic metallic state in the pressurized MnBi2Te4 single crystal. Phys. Rev. Mater. 2019, 3, 094201 10.1103/PhysRevMaterials.3.094201.

[ref20] Pei C.; Xia Y.; Wu J.; Zhao Y.; Gao L.; Ying T.; Gao B.; Li N.; Yang W.; Zhang D.; Gou H.; Chen Y.; Hosono H.; Li G.; Qi Y. Pressure-Induced Topological and Structural Phase Transitions in an Antiferromagnetic Topological Insulator. Chin. Phys. Lett. 2020, 37, 066401 10.1088/0256-307X/37/6/066401.

[ref21] Li T.; Jiang S.; Sivadas N.; Wang Z.; Xu Y.; Weber D.; Goldberger J. E.; Watanabe K.; Taniguchi T.; Fennie C. J.; Fai Mak K.; Shan J. Pressure-controlled interlayer magnetism in atomically thin CrI3. Nat. Mater. 2019, 18, 1303–1308. 10.1038/s41563-019-0506-1. 31659292

[ref22] Mankowsky R.; Först M.; Cavalleri A. Non-equilibrium control of complex solids by nonlinear phononics. Rep. Prog. Phys. 2016, 79, 064503 10.1088/0034-4885/79/6/064503. 27223639

[ref23] Oka T.; Kitamura S. Floquet Engineering of Quantum Materials. Annual Review of Condensed Matter Physics 2019, 10, 387–408. 10.1146/annurev-conmatphys-031218-013423.

[ref24] Rudner M. S.; Lindner N. H. Band structure engineering and non-equilibrium dynamics in Floquet topological insulators. Nature Reviews Physics 2020, 2, 229–244. 10.1038/s42254-020-0170-z.

[ref25] Rodriguez-Vega M.; Vogl M.; Fiete G. A. Low-frequency and Moiré–Floquet engineering: A review. Annals of Physics 2021, 435, 168434 10.1016/j.aop.2021.168434.

[ref26] Först M.; Manzoni C.; Kaiser S.; Tomioka Y.; Tokura Y.; Merlin R.; Cavalleri A. Nonlinear phononics as an ultrafast route to lattice control. Nat. Phys. 2011, 7, 854 10.1038/nphys2055.

[ref27] Subedi A.; Cavalleri A.; Georges A. Theory of nonlinear phononics for coherent light control of solids. Phys. Rev. B 2014, 89, 220301 10.1103/PhysRevB.89.220301.

[ref28] Fausti D.; Tobey R. I.; Dean N.; Kaiser S.; Dienst A.; Hoffmann M. C.; Pyon S.; Takayama T.; Takagi H.; Cavalleri A. Light-Induced Superconductivity in a Stripe-Ordered Cuprate. Science 2011, 331, 189–191. 10.1126/science.1197294. 21233381

[ref29] Mankowsky R.; et al. Nonlinear lattice dynamics as a basis for enhanced superconductivity in YBa2Cu3O6.5. Nature 2014, 516, 71 10.1038/nature13875. 25471882

[ref30] Mitrano M.; Cantaluppi A.; Nicoletti D.; Kaiser S.; Perucchi A.; Lupi S.; Di Pietro P.; Pontiroli D.; Riccò M.; Clark S. R.; Jaksch D.; Cavalleri A. Possible light-induced superconductivity in K3C60 at high temperature. Nature 2016, 530, 461 10.1038/nature16522. 26855424 PMC4820655

[ref31] Mankowsky R.; von Hoegen A.; Först M.; Cavalleri A. Ultrafast Reversal of the Ferroelectric Polarization. Phys. Rev. Lett. 2017, 118, 197601 10.1103/PhysRevLett.118.197601. 28548509

[ref32] Nova T. F.; Disa A. S.; Fechner M.; Cavalleri A. Metastable ferroelectricity in optically strained SrTiO3. Science 2019, 364, 1075–1079. 10.1126/science.aaw4911. 31197010

[ref33] Disa A. S.; Fechner M.; Nova T. F.; Liu B.; Först M.; Prabhakaran D.; Radaelli P. G.; Cavalleri A. Polarizing an antiferromagnet by optical engineering of the crystal field. Nat. Phys. 2020, 16, 937–941. 10.1038/s41567-020-0936-3.

[ref34] Stupakiewicz A.; Davies C. S.; Szerenos K.; Afanasiev D.; Rabinovich K. S.; Boris A. V.; Caviglia A.; Kimel A. V.; Kirilyuk A. Ultrafast phononic switching of magnetization. Nat. Phys. 2021, 17, 489–492. 10.1038/s41567-020-01124-9.

[ref35] Vaskivskyi I.; Gospodaric J.; Brazovskii S.; Svetin D.; Sutar P.; Goreshnik E.; Mihailovic I. A.; Mertelj T.; Mihailovic D. Controlling the metal-to-insulator relaxation of the metastable hidden quantum state in 1T-TaS2. Sci. Adv. 2015, 10.1126/sciadv.1500168. PMC4646782 26601218

[ref36] Stojchevska L.; Vaskivskyi I.; Mertelj T.; Kusar P.; Svetin D.; Brazovskii S.; Mihailovic D. Ultrafast Switching to a Stable Hidden Quantum State in an Electronic Crystal. Science 2014, 344, 177–180. 10.1126/science.1241591. 24723607

[ref37] Deng Y.; Yu Y.; Shi M. Z.; Guo Z.; Xu Z.; Wang J.; Chen X. H.; Zhang Y. Quantum anomalous Hall effect in intrinsic magnetic topological insulator MnBi2Te4. Science 2020, 367, 895–900. 10.1126/science.aax8156. 31974160

[ref38] Liu C.; Wang Y.; Li H.; Wu Y.; Li Y.; Li J.; He K.; Xu Y.; Zhang J.; Wang Y. Robust axion insulator and Chern insulator phases in a two-dimensional antiferromagnetic topological insulator. Nat. Mater. 2020, 19, 522–527. 10.1038/s41563-019-0573-3. 31907415

[ref39] Yang S.; Xu X.; Zhu Y.; Niu R.; Xu C.; Peng Y.; Cheng X.; Jia X.; Huang Y.; Xu X.; Lu J.; Ye Y. Odd-Even Layer-Number Effect and Layer-Dependent Magnetic Phase Diagrams in MnBi_2_Te_4_. Phys. Rev. X 2021, 11, 011003 10.1103/PhysRevX.11.011003.

[ref40] Yan J.-Q.; Okamoto S.; McGuire M. A.; May A. F.; McQueeney R. J.; Sales B. C. Evolution of structural, magnetic, and transport properties in MnBi_2–*x*_Sb_*x*_Te_4_. Phys. Rev. B 2019, 100, 104409 10.1103/PhysRevB.100.104409.

[ref41] Wimmer S.; et al. Mn-rich MnSb_2_Te_4_: A topological insulator with magnetic gap closing at high Curie temperatures of 45-50 K. Adv. Mater. 2021, 33, 2102935 10.1002/adma.202102935. PMC11468489 34469013

[ref42] Ge W.; Sass P. M.; Yan J.; Lee S. H.; Mao Z.; Wu W. Direct evidence of ferromagnetism in MnSb_2_Te_4_. Phys. Rev. B 2021, 103, 134403 10.1103/PhysRevB.103.134403.

[ref43] Rodriguez-Vega M.; Leonardo A.; Fiete G. A. Group theory study of the vibrational modes and magnetic order in the topological antiferromagnet MnBi_2_Te_4_. Phys. Rev. B 2020, 102, 104102 10.1103/PhysRevB.102.104102.

[ref44] Först M.; Mankowsky R.; Bromberger H.; Fritz D.; Lemke H.; Zhu D.; Chollet M.; Tomioka Y.; Tokura Y.; Merlin R.; Hill J.; Johnson S.; Cavalleri A. Displacive lattice excitation through nonlinear phononics viewed by femtosecond X-ray diffraction. Solid State Commun. 2013, 169, 24–27. 10.1016/j.ssc.2013.06.024.

[ref45] Nova T. F.; Cartella A.; Cantaluppi A.; Först M.; Bossini D.; Mikhaylovskiy R. V.; Kimel A. V.; Merlin R.; Cavalleri A. An effective magnetic field from optically driven phonons. Nat. Phys. 2017, 13, 132–136. 10.1038/nphys3925.

[ref46] Sentef M. A.; Kemper A. F.; Georges A.; Kollath C. Theory of light-enhanced phonon-mediated superconductivity. Phys. Rev. B 2016, 93, 144506 10.1103/PhysRevB.93.144506.

[ref47] Khalsa G.; Benedek N. A. Ultrafast optically induced ferromagnetic/anti-ferromagnetic phase transition in GdTiO3 from first principles. npj Quantum Mater. 2018, 3, 15 10.1038/s41535-018-0086-3.

[ref48] Juraschek D. M.; Fechner M.; Spaldin N. A. Ultrafast Structure Switching through Nonlinear Phononics. Phys. Rev. Lett. 2017, 118, 054101 10.1103/PhysRevLett.118.054101. 28211740

[ref49] Rodriguez-Vega M.; Lin Z.-X.; Leonardo A.; Ernst A.; Chaudhary G.; Vergniory M. G.; Fiete G. A. Phonon-mediated dimensional crossover in bilayer CrI_3_. Phys. Rev. B 2020, 102, 081117 10.1103/PhysRevB.102.081117.

[ref50] Gonze X.; Lee C. Dynamical matrices, Born effective charges, dielectric permittivity tensors, and interatomic force constants from density-functional perturbation theory. Phys. Rev. B 1997, 55, 10355–10368. 10.1103/PhysRevB.55.10355.

[ref51] Baroni S.; de Gironcoli S.; Dal Corso A.; Giannozzi P. Phonons and related crystal properties from density-functional perturbation theory. Rev. Mod. Phys. 2001, 73, 515–562. 10.1103/RevModPhys.73.515.

[ref52] Choe J.; Lujan D.; Rodriguez-Vega M.; Ye Z.; Leonardo A.; Quan J.; Nunley T. N.; Chang L.-J.; Lee S.-F.; Yan J.; Fiete G. A.; He R.; Li X. Electron–Phonon and Spin–Lattice Coupling in Atomically Thin Layers of MnBi2Te4. Nano Lett. 2021, 21, 6139 10.1021/acs.nanolett.1c01719. 34252281

[ref53] Guo W.-T.; Huang L.; Yang Y.; Huang Z.; Zhang J.-M. Pressure-induced topological quantum phase transition in the magnetic topological insulator MnBi2Te4. New J. Phys. 2021, 23, 083030 10.1088/1367-2630/ac1974.

[ref54] Sell A.; Leitenstorfer A.; Huber R. Phase-locked generation and field-resolved detection of widely tunable terahertz pulses with amplitudes exceeding 100 MV/cm. Opt. Lett. 2008, 33, 2767–2769. 10.1364/OL.33.002767. 19037420

[ref55] Kampfrath T.; Tanaka K.; Nelson K. A. Resonant and nonresonant control over matter and light by intense terahertz transients. Nat. Photonics 2013, 7, 680 10.1038/nphoton.2013.184.

[ref56] Li B.; Yan J.-Q.; Pajerowski D.; Gordon E.; Nedić A.-M.; Sizyuk Y.; Ke L.; Orth P.; Vaknin D.; McQueeney R. Competing Magnetic Interactions in the Antiferromagnetic Topological Insulator MnBi2Te4. Phys. Rev. Lett. 2020, 124, 167204 10.1103/PhysRevLett.124.167204. 32383954

[ref57] Liechtenstein A. I.; Katsnelson M. I.; Antropov V. P.; Gubanov V. A. Local spin density functional approach to the theory of exchange interactions in ferromagnetic metals and alloys. J. Magn. Magn. Mater. 1987, 67, 65–74. 10.1016/0304-8853(87)90721-9.

[ref58] Hoffmann M.; Ernst A.; Hergert W.; Antonov V. N.; Adeagbo W. A.; Geilhufe R. M.; Ben Hamed H. Magnetic and Electronic Properties of Complex Oxides from First-Principles. Phys. Status Solidi B 2020, 257, 1900671 10.1002/pssb.201900671.

[ref59] Lai Y.; Ke L.; Yan J.; McDonald R. D.; McQueeney R. J. Defect-driven ferrimagnetism and hidden magnetization in MnBi2Te4. Phys. Rev. B 2021, 103, 184429 10.1103/PhysRevB.103.184429.

[ref60] Granado E.; García A.; Sanjurjo J. A.; Rettori C.; Torriani I.; Prado F.; Sánchez R. D.; Caneiro A.; Oseroff S. B. Magnetic ordering effects in the Raman spectra of La_1–*x*_Mn_1–*x*_O_3_. Phys. Rev. B 1999, 60, 11879–11882. 10.1103/PhysRevB.60.11879.

[ref61] Gilbert T. A phenomenological theory of damping in ferromagnetic materials. IEEE Trans. Magn. 2004, 40, 3443–3449. 10.1109/TMAG.2004.836740.

[ref62] Yan J.-Q.; Zhang Q.; Heitmann T.; Huang Z.; Chen K. Y.; Cheng J.-G.; Wu W.; Vaknin D.; Sales B. C.; McQueeney R. J. Crystal growth and magnetic structure of MnBi2Te4. Phys. Rev. Materials 2019, 3, 064202 10.1103/PhysRevMaterials.3.064202.

[ref63] Liu Y.; Wang L.-L.; Zheng Q.; Huang Z.; Wang X.; Chi M.; Wu Y.; Chakoumakos B. C.; McGuire M. A.; Sales B. C.; Wu W.; Yan J. Site Mixing for Engineering Magnetic Topological Insulators. Phys. Rev. X 2021, 11, 021033 10.1103/PhysRevX.11.021033.

[ref64] Yuan Y.; Wang X.; Li H.; Li J.; Ji Y.; Hao Z.; Wu Y.; He K.; Wang Y.; Xu Y.; Duan W.; Li W.; Xue Q.-K. Electronic States and Magnetic Response of MnBi2Te4 by Scanning Tunneling Microscopy and Spectroscopy. Nano Lett. 2020, 20, 3271–3277. 10.1021/acs.nanolett.0c00031. 32298117

[ref65] Li H.; Liu S.; Liu C.; Zhang J.; Xu Y.; Yu R.; Wu Y.; Zhang Y.; Fan S. Antiferromagnetic topological insulator MnBi2Te4: synthesis and magnetic properties. Phys. Chem. Chem. Phys. 2020, 22, 556–563. 10.1039/C9CP05634C. 31840700

[ref66] Gyorffy B. L. Coherent-Potential Approximation for a Nonoverlapping-Muffin-Tin-Potential Model of Random Substitutional Alloys. Phys. Rev. B 1972, 5, 2382–2384. 10.1103/PhysRevB.5.2382.

[ref67] Vergniory M. G.; Otrokov M. M.; Thonig D.; Hoffmann M.; Maznichenko I. V.; Geilhufe M.; Zubizarreta X.; Ostanin S.; Marmodoro A.; Henk J.; Hergert W.; Mertig I.; Chulkov E. V.; Ernst A. Exchange interaction and its tuning in magnetic binary chalcogenides. Phys. Rev. B 2014, 89, 165202 10.1103/PhysRevB.89.165202.

[ref68] Otrokov M.; Rusinov I.; Blanco-Rey M.; Hoffmann M.; Vyazovskaya A.; Eremeev S.; Ernst A.; Echenique P.; Arnau A.; Chulkov E. Unique Thickness-Dependent Properties of the van der Waals Interlayer Antiferromagnet MnBi_2_Te_4_ Films. Phys. Rev. Lett. 2019, 122, 107202 10.1103/PhysRevLett.122.107202. 30932645

[ref69] Bansil A.; Lin H.; Das T. Colloquium: Topological band theory. Rev. Mod. Phys. 2016, 88, 021004 10.1103/RevModPhys.88.021004.

