# Supplementary material for: Light-Driven Topological and Magnetic Phase Transitions in Thin Layer Antiferromagnets
Source: J Phys Chem Lett. 2022 May 4;13(18):4152–8. doi: 10.1021/acs.jpclett.2c00070 (PMC9109223; doi:10.1021/acs.jpclett.2c00070)
Supplement: Supplementary file 1 — jz2c00070_si_001.pdf [file jz2c00070_si_001.pdf]

Supporting Information: "Light-driven Topological and Magnetic Phase Transitions in Thin-layer Antiferromagnets"

Martin Rodriguez-Vega,[†] Ze-Xun Lin,[‡,¶] Aritz Leonardo,[§,∥] Arthur Ernst,[⊥,#] Maia G. Vergniory,[§,@] and Gregory A. Fiete[¶,△]

†*Theoretical Division, Los Alamos National Laboratory, Los Alamos, New Mexico 87545, USA*

‡*Department of Physics, The University of Texas at Austin, Austin, TX 78712, USA*

¶*Department of Physics, Northeastern University, Boston, MA 02115, USA*

§*Donostia International Physics Center, Paseo Manuel de Lardizabal 4, 20018 San Sebastian, Spain*

∥*Department of Physics, University of the Basque Country UPV/ EHU, Leioa, Spain.*

⊥*Institut für Theoretische Physik, Johannes Kepler Universität, A 4040 Linz, Austria*

#*Max-Planck-Institut für Mikrostrukturphysik, Weinberg 2, D-06120 Halle, Germany*

@*Max Planck Institute for Chemical Physics of Solids, Dresden, D-01187, Germany*

△*Department of Physics, Massachusetts Institute of Technology, Cambridge, MA 02139, USA*

E-mail:

Phonon symmetry analysis details

The projection operators used to block-diagonalize the dynamical matrix are defined as[1,2]

$$\hat{P}_{kl}^{(\Gamma_n)} = \frac{l_n}{h} \sum_{C_\alpha} \left(D_{kl}^{(\Gamma_n)}(C_\alpha) \right)^* \hat{P}(C_\alpha), \tag{1}$$

where $\hat{P}_{kl}^{(\Gamma_n)}$, Γ_n labels the irreducible representations (irreps) in the point group, C_α corresponds to the elements of the group, $D_{kl}^{(\Gamma_n)}(C_\alpha)$ is the irreducible matrix representation of C_α, h is the order of D_{3d}, and l_n is the dimension of the irreducible representation Γ_n. $\hat{P}(C_\alpha)$ are $3N \times 3N$ matrices that form the displacement representation. We employ the software suite ISODISTORT to calculate the lattice displacements.

D_{3d} point group Character table

In this section, we list the character table for point group considered in this work.

Table 1: D_{3d} point group character table.

D_{3d}	E	$2C_3$	$3C_2'$	i	$2S_6$	$3\sigma_d$	functions
A_{1g}	1	1	1	1	1	1	x^2+y^2, z^2
A_{2g}	1	1	-1	1	1	-1	
E_g	2	-1	0	2	-1	0	x^2-y^2, xy, xz,yz
A_{1u}	1	1	1	-1	-1	-1	
A_{2u}	1	1	-1	-1	-1	1	z
E_u	2	-1	0	-2	1	0	x, y

Phonon computational details

We employ finite difference methods as implemented in VASP. We introduce a 16 Angstrom vacuum between the 2-SLs employing the generalized gradient approximation with PAW pseudo-potentials with an energy cut-off of 700 eV, a 13x13x1 k-point Monkhorst pack mesh, Gaussian smearing with 0.005eV width, and the van der Waals corrections DFT-D3 method of Grimme. We perform a non-collinear calculation in the AFM configuration with SOC included. The relaxation criteria

established a total force on each atom equal to or less than 0.1 meV/Angstrom. The parameter U=5.3 eV is introduced for the Mn atoms.[3]

Born effective charges

The Born effective charges tensors $Z^{*}_{\kappa,ij}$ are calculated with VASP. The results for MBT, in units of the electron charge and in Cartesian coordinates, are

Te	1		
1	−0.63084	−0.01998	−0.01636
2	−1.33825	−2.28334	3.32695
3	0.00000	0.00000	8.80461
Te	2		
1	−0.77164	−0.03543	0.02245
2	−0.52552	−1.12475	−7.71475
3	−0.00000	0.00000	−0.23496
Te	3		
1	−1.62824	0.02172	0.01599
2	0.14163	−4.13173	1.73877
3	0.00000	−0.00000	0.74167
Te	4		
1	−1.08403	0.00561	−0.02381
2	−0.38649	−2.73453	5.59597
3	0.00000	−0.00000	−2.03785
Te	5		
1	−1.08403	−0.00561	0.02381
2	−0.28895	−2.79751	5.63415
3	0.00000	0.00000	−2.06438
Te	6		
1	−1.62824	−0.02172	−0.01599
2	0.20170	−4.06610	1.73826
3	0.00000	−0.00000	0.72573
Te	7		
1	−0.77164	0.03543	−0.02245
2	−0.87899	−1.03579	−7.93368
3	0.00000	−0.00000	−0.44477
Te	8		
1	−0.63084	0.01998	0.01636
2	−1.34101	−2.24510	3.42555
3	0.00000	−0.00000	8.75698

Bi	11		
1	2.06710	0.03874	−0.00257
2	−1.80210	21.52997	12.18834
3	−0.00000	0.00000	−2.50140
Bi	12		
1	2.65096	0.01958	0.02168
2	4.71818	6.77268	12.20400
3	−0.00000	0.00000	−4.12207
Bi	13		
1	2.65096	−0.01958	−0.02168
2	5.28712	6.63998	12.14577
3	−0.00000	0.00000	−4.02684
Bi	14		
1	2.06710	−0.03874	0.00257
2	−1.52371	20.73211	11.92867
3	0.00000	−0.00000	−2.11469

The Born effective charge for phonon mode α is defined as[4,5]

$$Z^*_{\alpha,i} = \sum_{\kappa,j} Z^*_{\kappa,ij} \frac{e_{\alpha,\kappa,j}}{\sqrt{m_\kappa}}, \tag{2}$$

where i the direction in Cartesian coordinates, the index κ labels the atoms in the unit cell, m_κ is the mass of the κ atom, and $e_{\alpha,\kappa,j}$ are the normalized dynamical matrix eigenvectors. The Born effective charge for 2-SL MBT and 2-SL MST are shown in Figs. 1 and 2 .

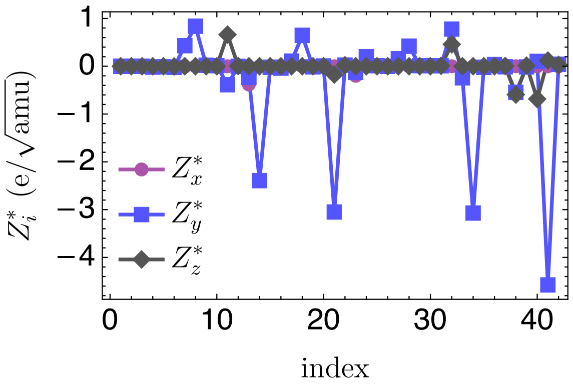

Figure 1: Born effective charge for 2-SL MBT phonon modes, indexed from largest to smallest frequency.

For 2-SL MST, we find the following Born effective charges tensors $Z^*_{\kappa,ij}$

Te	1		
1	−0.94933	0.00971	−0.00817
2	0.15368	−0.89636	−0.29914
3	0.00000	−0.00000	−0.61124
Te	2		
1	−1.05613	−0.01486	−0.00691
2	0.37929	−1.33225	−2.23013
3	−0.00000	0.00000	1.65176
Te	3		
1	−1.85390	0.01480	−0.00371
2	−0.03114	−1.94616	−1.58089
3	0.00000	−0.00000	0.43603
Te	4		
1	−1.08178	−0.01610	−0.03037
2	−0.08758	−1.32154	−0.19687
3	−0.00000	−0.00000	−0.01256
Te	5		
1	−1.08178	0.01610	0.03037
2	−0.09951	−1.31466	−0.15934
3	0.00000	−0.00000	−0.03296
Te	6		
1	−1.85390	−0.01480	0.00371
2	−0.03295	−1.92913	−1.63727
3	0.00000	−0.00000	0.40742
Te	7		
1	−1.05613	0.01486	0.00691
2	0.34676	−1.32141	−2.22054
3	−0.00000	0.00000	1.62904

Te	8		
1	−0.94933	−0.00971	0.00817
2	0.18951	−0.89104	−0.25157
3	0.00000	0.00000	−0.65512
Mn	9		
1	−0.30459	0.00643	0.03869
2	0.08618	−1.50016	0.15385
3	−0.00000	−0.00000	−0.99898
Mn	10		
1	−0.30459	−0.00643	−0.03869
2	0.09135	−1.45618	0.15236
3	0.00000	−0.00000	−0.98569
Sb	11		
1	2.45609	−0.00188	0.01025
2	−0.44205	3.47813	1.97963
3	0.00000	0.00000	0.76708
Sb	12		
1	2.78963	0.01087	0.02941
2	−0.04583	3.50654	2.16076
3	0.00000	−0.00000	−1.21288
Sb	13		
1	2.78963	−0.01087	−0.02941
2	−0.03490	3.47281	2.16225
3	−0.00000	−0.00000	−1.17246
Sb	14		
1	2.45609	0.00188	−0.01025
2	−0.47280	3.45141	1.96690
3	0.00000	−0.00000	0.79057

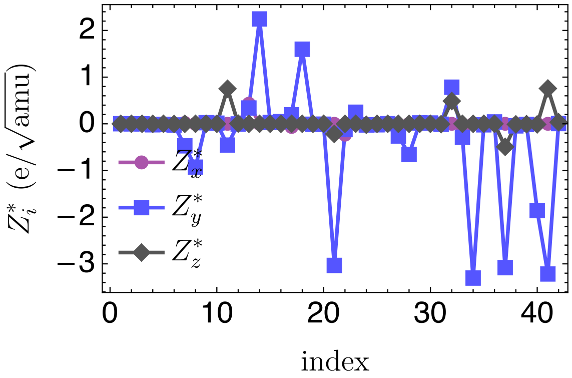

Figure 2: Born effective charge for 2-SL MST phonon modes, indexed from largest to smallest frequency.

Non-linear coefficients

In this section, we provide the non-linear coefficients for the energy potentials discussed in the main text.

Table 2: Non-linear coefficients for 2-SL MBT and MST.

2-SL MBT	
γ_3	-2.9×10^{-5}eV/(Å$\sqrt{\text{amu}}$)^3
β_3	-3.5×10^{-5}eV/(Å$\sqrt{\text{amu}}$)^3
2-SL MST	
$\gamma_{1,3}$	-2.8×10^{-5}eV/(Å$\sqrt{\text{amu}}$)^3
$\gamma_{2,3}$	-4.2×10^{-5}eV/(Å$\sqrt{\text{amu}}$)^3
$\gamma_{1,2,3}$	-4.6×10^{-5}eV/(Å$\sqrt{\text{amu}}$)^3
β_3	-4.0×10^{-5}eV/(Å$\sqrt{\text{amu}}$)^3

Single-particle electronic excitation spectrum

In this section, we calculate the single-particle electronic excitation spectrum for 2SL-MBT. We employ the low-energy Hamiltonian introduced in Ref.,[6] given by

$$H = \sum_{\mathbf{k}_\perp, ij} \left[\left((-)^i \hbar v_D (\hat{z} \times \boldsymbol{\sigma}) \cdot \mathbf{k}_\perp + m_i \sigma_z \right) \delta_{ij} \; + \Delta_{ij} \left(1 - \delta_{ij}\right) \right] c^\dagger_{\mathbf{k}_\perp i} c_{\mathbf{k}_\perp j} = \sum_{\mathbf{k}_\perp, ij} H_{ij}(\mathbf{k}_\perp) c^\dagger_{\mathbf{k}_\perp i} c_{\mathbf{k}_\perp j}. \tag{3}$$

The first term describes the Dirac cones at the surfaces of the thin films (top and bottom), v_D is the Fermi velocity of the surface fermions, $\mathbf{k}_\perp$ describe the in-plane momenta, and $\boldsymbol{\sigma}$ are the Pauli matrices in spin space. The indices i,j run over the surfaces. The second term describes the effect of coupling of the electronic states with the Mn magnetic moments with $m_i = \sum_\alpha J_{i\alpha} M_\alpha$, where α labels the layers, i the Dirac cones, and $M_\alpha = \pm 1$ for magnetic layers. Finally, the third term, Δ_{ij}, is the hopping amplitude between the i-th and j-th Dirac cones. The parameters $\Delta_S = 84$ meV, $\Delta_D = -127$ meV $J_S = 36$ meV, $J_D = 29$ meV for same (S) and different (D) layers, corresponds to $MnBi_2Te_4$.[6] In Fig. 3, we plot the energy bands before laser excitation.

Assuming that the laser pulse is linearly polarized, the time-dependent Hamiltonian is given by $H(t) = H(\mathbf{k}_\perp(t))$, where $k_x \to k_x - A \sin(\Omega t) f(t)$, $f(t) = \exp\{-t^2/(2\sigma^2)\}$, and σ is the standard

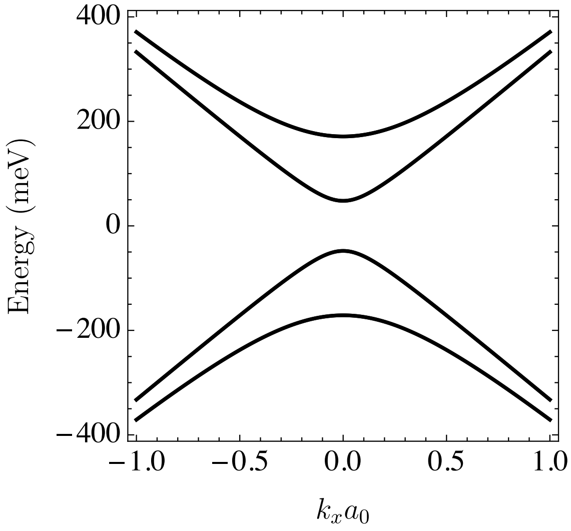

Figure 3: Energy bands as a function of momentum k_x and $k_y = 0$ obtain by diagonalizing the low-energy Hamiltonian Eq. (3). The model parameters are $\Delta_S = 84$ meV, $\Delta_D = -127$ meV $J_S = 36$ meV, and $J_D = 29$ meV.

deviation (width in time) of the pulse.

We are interested in the probability P of finding the system in an excited state as a consequence of the laser excitation,[7]

$$P(t, \mathbf{k}_\perp) = |\langle \psi(t, \mathbf{k}_\perp)|\psi(0, \mathbf{k}_\perp)\rangle|^2, \tag{4}$$

where $|\psi(0, \mathbf{k}_\perp)\rangle$ is the initial Bloch state, and

$$|\psi(t, \mathbf{k}_\perp)\rangle = U(t, \mathbf{k}_\perp)|\psi(0, \mathbf{k}_\perp)\rangle, \tag{5}$$

where $U(t, \mathbf{k}_\perp) = T \exp\{-i \int_0^t H(\mathbf{k}_\perp(s))ds\}$ is the time-ordered propagator, which we evaluate numerically employing a Trotter approximation.

We also consider the effect of weak interactions by introducing a phenomenological self-energy term Σ. The Schrödinger equation takes the form $\mathrm{i}\hbar \frac{\mathrm{d}}{\mathrm{dt}}|\psi(t)\rangle = (H(t) - i\Sigma)|\psi(t)\rangle$, and one can still construct the propagator $U(t, t')$. Other approaches can be employed, such as the (t, t') approach[8] and its extension to non-Hermitian systems.[9,10] We choose $\Sigma = \gamma \mathbf{1}$, where $\mathbf{1}$ is the identity in our model, and γ is the spectral broadening.

In Fig. 4 we plot the time average of $P(t, \mathbf{k}_\perp = 0)$ in a time interval that captures the

duration of the pulse as a function of the laser intensity $A = ea_0 E_0/(\hbar\Omega_{IR})$, with laser frequency $\Omega_{IR}/(2\pi) \approx 5$ THz. As the laser intensity increases, the probability to remain in the initial state decreases. However, for $A = 3.5$, which corresponds to the peak electric field $E_0 \approx 1.7$ MV/cm, the average probability is still ~ 0.7. Weak interactions do not change drastically the time-averaged $P(t, \mathbf{k}_\perp = 0)$. This toy model indicates that materials that require smaller peak electric field are better suited to minimize single-particle excitations.

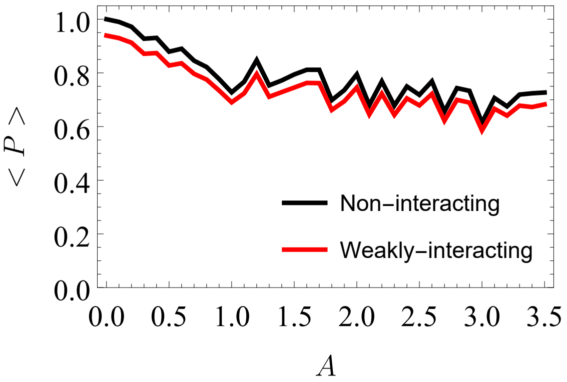

Figure 4: Time average probability to occupy the initial Bloch state as a function of the laser intensity A, for the non-interacting (black) and weakly-interacting cases (red). The electronic spectral broadening is assumed to be 0.05 meV (red curve). The rest of the parameters are the same as in Fig. 3.

References

(1) Dresselhaus, M. S.; Dresselhaus, G.; Jorio, A. *Group theory: Application to the Physics of Condensed Matter*; Springer-Verlag Berlin Heidelberg, 2008.

(2) Hergert, W.; Geilhufe, M. R. *Group theory in solid state physics and photonics: problem solving with Mathematica*; Wiley-VCH: Weinheim, 2018.

(3) Otrokov, M. M. et al. Prediction and observation of an antiferromagnetic topological insulator. *Nature* **2019**, *576*, 416–422.

(4) Gonze, X.; Lee, C. Dynamical matrices, Born effective charges, dielectric permittivity tensors, and interatomic force constants from density-functional perturbation theory. *Phys. Rev. B* **1997**, *55*, 10355–10368.

(5) Baroni, S.; de Gironcoli, S.; Dal Corso, A.; Giannozzi, P. Phonons and related crystal properties from density-functional perturbation theory. *Rev. Mod. Phys.* **2001**, *73*, 515–562.

(6) Lei, C.; Chen, S.; MacDonald, A. H. Magnetized topological insulator multilayers. *Proceedings of the National Academy of Sciences* **2020**, *117*, 2722427230.

(7) Strter, C.; Eckardt, A. Interband Heating Processes in a Periodically Driven Optical Lattice. *Zeitschrift fr Naturforschung A* **2016**, *71*, 909920.

(8) Peskin, U.; Moiseyev, N. The solution of the timedependent Schrdinger equation by the (t,t) method: Theory, computational algorithm and applications. *The Journal of Chemical Physics* **1993**, *99*, 4590–4596.

(9) Fleischer, A.; Moiseyev, N. Adiabatic theorem for non-Hermitian time-dependent open systems. *Phys. Rev. A* **2005**, *72*, 032103.

(10) Gilary, I.; Fleischer, A.; Moiseyev, N. Calculations of time-dependent observables in non-Hermitian quantum mechanics: The problem and a possible solution. *Phys. Rev. A* **2005**, *72*, 012117.
