# Supplementary material for: Light-Driven Topological and Magnetic Phase Transitions in Thin Layer Antiferromagnets
Source: J Phys Chem Lett. 2022 May 4;13(18):4152–8. doi: 10.1021/acs.jpclett.2c00070 (PMC9109223; doi:10.1021/acs.jpclett.2c00070)
Supplement: Supplementary file 2 — jz2c00070_si_002.pdf [file jz2c00070_si_002.pdf]

jz-2022-00070t.R1

Name: Peer Review Information for "Light-driven Topological and Magnetic Phase Transitions in Thin-layer Antiferromagnets"

First Round of Reviewer Comments

Reviewer: 1

Comments to the Author

In the manuscript of “Light-driven Topological and Magnetic Phase Transitions in Thin-layer Antiferromagnets”, the authors have investigated theoretically that nonlinear phononics driven by the intense infrared laser excitation could modify the magnetic order in antiferromagnetic topological insulators under the non-equilibrium condition. The authors argue that this work could provide an additional mechanism to achieve magneto-topological transitions without applying magnetic field or pressure. Overall, this research is interesting and can be considered for publish after addressing the following comments.

1. It is well known that phonon amplitude is damping with time after photon excitation. In the calculation, all the nonlinear interaction potential did not include the damping effect (or phonon lifetime), please comment on how phonon damping will affect the non-equilibrium magneto-topological transitions.

2. In Page 6, it is hard to understand why nonlinear coupling between IR phonon (A2u) and Raman-active breathing phonon (A1g) can only lead to an increase in the Mn-Mn layer separation since ‘breathing mode’ can be either symmetric (increase the layer distance) and anti-symmetric (decrease the layer distance).

3. In page 8, the authors said anharmonic lattice oscillations (<u> ≠ 0) can renormalize the phonon-spin interaction, thus leading to different magnetic order. Please evaluate the intrinsic timescale of this renormalization process, does it comparable to the phonon lifetime?

4. For the disorder effect, the anti-site disorder is introduced to the Mn layer or the Bi(Sb) layer? and will it make any difference to the modelling result?

Reviewer: 2

Comments to the Author

This paper shows that two septuple-layer(2-SL) MnBi2Te4(MBT) and MnSb2Te4(MST) can provide excellent theoretical platforms to study the effects of non-equilibrium magneto-

topological order via non-linear interactions with Raman phonon modes induced by photo-excited infrared phonons.

First, from symmetry analysis, moderate laser intensities attainable in current experimental setups can induce non-linear dynamics in the Raman breathing phonon mode. And the time-average of the phonon dynamics leads to effective lattice distortions that facilitate the change of magnetic order accompanied by topological transitions. In terms of the magnetic changes, the increased distances between magnetic atomic planes under the laser-induced structure contribute to the change of magnetic interactions. Furthermore, the magnetic order can be tuned by the anti-site disorder. On the other hand, the topological order of the new laser-induced structures appears in a form of the band inversion.

In a word, it highlights the order parameters that can be changed with non-linear phonon interactions indirectly by externally pumping an infrared (IR) -active phonon mode to modulate the equilibrium of the structure into a transient state of modified orders. Besides, it provides an initiative for more detailed experimental investigations to explore light controlled orders .

Before the paper can be judged for publication, a major revision is needed. Some technical suggestions are listed below:

1. In equation 3, the spin exchange interaction J is only related to displacement $\mu(t)$. This is only true using Born-Oppenheimer approximation. When the system illuminated with a laser, there are some non-adiabatic effects. Is Born-Oppenheimer approximation appropriate in this case?

2. In Fig. 3, $\langle Q_{(R(3))} \rangle$ is ranged from 0 to 5 Å√amu. Why does $\langle Q_{(R(3))} \rangle$ range from -10 to 10 Å√amu in Fig. 4?

3. Is E_0 the electric field E_l in the laser or $E_l/\varepsilon(\omega)$, where $\varepsilon(\omega)$ is the dielectric function under a laser with frequency ω?

4. How many unit cells have been used in this article? What's the meaning of N in equation $\tilde{J}_{eff}=1/N\sum_{ij} J_{ij}$?

5. How did the authors define the coupling constant δJ by Floquet theory?

6. What is the time scale of the spin flipping during magnetic phase transition? Is the time scale shorter than the phonon's lifetime? i.e. Can the phase transition be observed by experiments?

7. In Fig. 5, what does band inversion mean? What's the meaning of the bands' colors?

8. Supplementary information and Appendix are absent.

Author's Response to Peer Review Comments:

February 26, 2022

Dear Prof. Editor,

We are resubmitting our manuscript entitled “Light-driven topological and mag- netic phase transitions in thin-layer antiferromagnets” for further consideration in The Journal of Physical Chemistry Letters.

We thank you for your time handling our manuscript, and the referees for their detailed reports. In the revised version of the manuscript, we address all the referee concerns, which helped us improve our work. In our view, the main concerns of the referees are related to (i) the validity of our approximations, and
(ii) the possibility to observe the transition from antiferromagnet to ferromagnet given that phonons have a finite lifetime.

To support the validity of our approximations, we added a new section in the supplemental material. In essence, the short duration of the laser pulses re- quired, and the electronic gap in equilibrium (larger than the phonon frequen- cies) supports our approximations. To support the viability of observing the spin transition following laser excitation, we solved the Landau-Lifshitz-Gilbert equations. We found that the time scale for the transition is in the few to several picoseconds range, depending on the unknown parameters (we assume realistic values), such as the Gilbert damping. In some cases, the time for spin flip is comparable to the phonon lifetime. However, as we point out in the response, suitable train pulses can be engineering to prolong the phonon effect. Therefore, we sustain that the phonon-induced antiferromagnet to ferromagnet transition could be observed in experiments.

We hope you agree that the revised version of our work is suitable for publication in your esteemed journal.

Sincerely,

The authors

Reply to Referees.

Reviewer: 1

Recommendation: This paper is publishable subject to minor revisions noted. Further review is not needed.

We thank the referee for their detailed read of our manuscript. Below we address the questions raised by the referee.

Comments:

In the manuscript of “Light-driven Topological and Magnetic Phase Transitions in Thin-layer Antiferromagnets”, the authors have investigated theoretically that nonlinear phononics driven by the intense infrared laser excitation could modify the magnetic order in antiferromagnetic topological insulators under the non-equilibrium condition. The authors argue that this work could provide an additional mechanism to achieve magneto-topological transitions without applying magnetic field or pressure. Overall, this research is interesting and can be considered for publish after addressing the following comments.

1. It is well known that phonon amplitude is damping with time after photon excitation. In the calculation, all the nonlinear interaction potential did not include the damping effect (or phonon lifetime), please comment on how phonon damping will affect the non-equilibrium magneto-topological transitions.

The phonon lifetime establishes the duration of the laser-induced lattice distortion following laser excitation using ultra-short laser pulses (see, for example, Nature Physics 17, 489–492 (2021), reference 34 in the main text). According to experiments reported in Nano Letters 21 (14), 6139-6145 (2021), the lifetime of the A1g phonon we study here is ~13.3 ps (See figure 2 panel c in the Nano Letters). Therefore, we expect the transition to last for the phonon lifetime.

To address this question, we have added a sentence in the text, indicating the measured lifetime of the A1g phonon. (Shown in blue just below Eq (2).)

2. In Page 6, it is hard to understand why nonlinear coupling between IR phonon (A2u) and Raman-active breathing phonon (A1g) can only lead to an increase in the Mn-Mn layer separation since ‘breathing mode’ can be either symmetric (increase the layer distance) and anti-symmetric (decrease the layer distance).

We thank the referee for this question, which allowed us to clarify our discussion. As the referee indicates, the harmonic excitation of the breathing mode would lead to oscillations that increase and decrease the layer distance, with an average equal to the equilibrium interlayer distance. However, the non-linear interaction with the infrared mode leads to anharmonic oscillations

around a shifted equilibrium position. This new equilibrium position can only increase the layer distance, as determined by the sign of the anharmonic term coefficient (gamma_3 in Eq. (1) of the main text for MBT and gamma_13, gamma_23 in Eq. (2) of the main text for MST). This coefficient is extracted from our density functional theory calculations and is an intrinsic property of the material.

To address this issue, we added a sentence (shown in blue) on page 7.

3. In page 8, the authors said anharmonic lattice oscillations (<u> ≠ 0) can renormalize the phonon-spin interaction, thus leading to different magnetic order. Please evaluate the intrinsic timescale of this renormalization process, does it comparable to the phonon lifetime?

Before the laser arrives at the sample, the electronic gap is larger than the phonon frequencies. Therefore, we can assume that the electrons follow the motion of the ions adiabatically since electrons typically have dynamics in the femtosecond regime, much shorter than the phonon lifetime (~13.3 ps). The renormalization process of the exchange interaction is thus much shorter than the phonon lifetime.

Once we obtained J_eff, as given in Fig 4, we can assume that the magnetic moments follow the Landau-Lifshitz-Gilbert equations. We estimated the time evolution of the spin aligned antiferromagnetically under the new J_eff. Here we show an example for reasonable parameters.

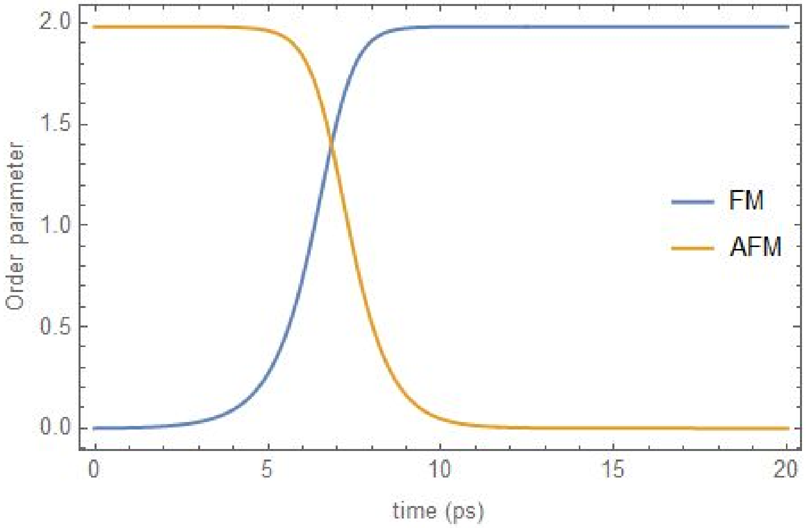

By changing the unknown parameters (such as the Gilbert damping factor, the spin anisotropy (Phys. Rev. Lett. 124, 167204 (2020))) within reason, the transition can be shifted down to 4 ps, or increased to 10 ps. The regime is indeed comparable to the phonon lifetime and can happen within it.

However, recent experiments (Nature Physics 17, 489–492 (2021)) demonstrating magnetization reversal via ultrafast phononics have shown that trains of lasers can be created in the laboratory. In principle, well-timed train pulses could sustain the distortion for longer than the phonon lifetime. In such a case, the limit would be imposed by the threshold for sample laser damage.

To address this comment, we added a sentence on page 9, shown in blue.

4. For the disorder effect, the anti-site disorder is introduced to the Mn layer or the Bi(Sb) layer? and will it make any difference to the modeling result?

The anti-site disorder is assumed to be an interchange of Mn with Bi(Sb) elements between the Mn layer and Bi(Sb) layers. This is consistent with recent experiments (Adv. Mater. 33, 2102935 (2021)). Anti-site disorder effects were found to be very important for the exchange interaction in these materials, but not for phonons.

This can be understood within a simple model of phonons in two coupled one-dimensional atom chains with a two-point basis. The equations of motion are given by

$$
\begin{aligned}
M_1\partial_t^2 u_n^{(1,1)} &= -\kappa\left(2u_n^{(1,1)} - u_n^{(2,1)} - u_{n-1}^{(2,1)}\right) - \kappa_\perp\left(u_n^{(1,1)} - u_n^{(1,2)}\right)\\
M_2\partial_t^2 u_n^{(2,1)} &= -\kappa\left(2u_n^{(2,1)} - u_{n+1}^{(1,1)} - u_n^{(1,1)}\right) - \kappa_\perp\left(u_n^{(2,1)} - u_n^{(2,2)}\right)\\
M_2\partial_t^2 u_n^{(1,2)} &= -\kappa\left(2u_n^{(1,2)} - u_n^{(2,2)} - u_{n-1}^{(2,2)}\right) - \kappa_\perp\left(u_n^{(1,2)} - u_n^{(1,1)}\right)\\
M_2\partial_t^2 u_n^{(2,2)} &= -\kappa\left(2u_n^{(2,2)} - u_{n+1}^{(1,2)} - u_n^{(1,2)}\right) - \kappa_\perp\left(u_n^{(2,2)} - u_n^{(2,1)}\right)
\end{aligned}
$$

Where M1 and M2 are the masses of the two atoms on the two-point basis, k is the effective intra-layer coupling, and k_perp is the inter-layer coupling. u_n^(i,l) corresponds to the displacement of the n-th atom in sublattice i=1,2, and layer l=1,2 (top and bottom).

In the plot below, we show the phonon energies obtained in chains with 40 atoms in each layer. The blue dots correspond to a pristine sample, while the yellow dots correspond to a sample with 5% variation in the constants k, k_perp that simulate the disorder. We consider 200 disorder realizations. The variation of the energies is typically small, except for the lowest-frequency optical mode (phonon index = 3), corresponding to a shear mode where the layers slide left and right with respect to each other, as shown below. The shear phonon mode does not participate in the renormalization of the exchange interaction, due to symmetry constraints, as discussed in the main text. This simple model supports the experimental observations in 2-SL MBT, as discussed in the main text.

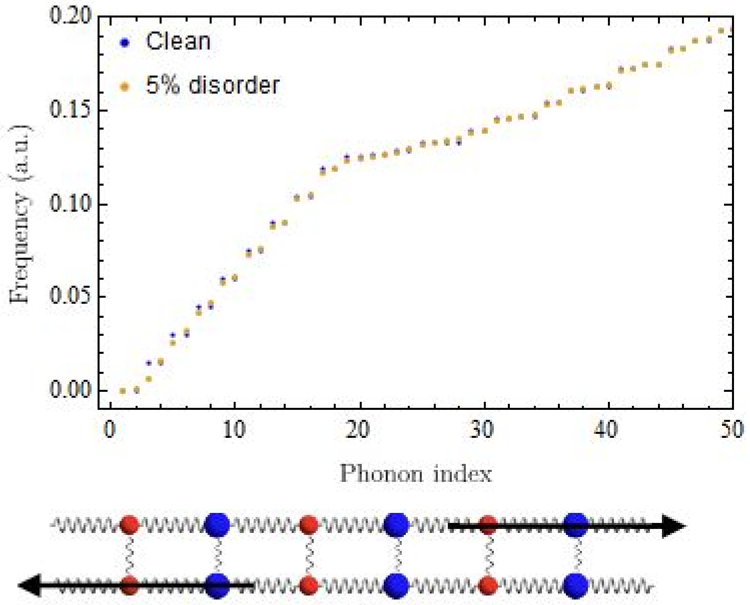

To address this point, we added the discussion on anti-site disorder on the first paragraph of page 10, shown in blue.

Reviewer: 2

Recommendation: This paper may be publishable, but major revision is needed; I would like to be invited to review any future revision.

We thank the referee for their detailed read of our manuscript. Below we address the questions raised by the referee. We hope the referee supports the publication of the revised version of the manuscript.

Comments:
This paper shows that two septuple-layer(2-SL) MnBi2Te4(MBT) and MnSb2Te4(MST) can provide excellent theoretical platforms to study the effects of non-equilibrium magneto-topological order via non-linear interactions with Raman phonon modes induced by photo-excited infrared phonons.

First, from symmetry analysis, moderate laser intensities attainable in current experimental setups can induce non-linear dynamics in the Raman breathing phonon mode. And the time-

average of the phonon dynamics leads to effective lattice distortions that facilitate the change of magnetic order accompanied by topological transitions. In terms of the magnetic changes, the increased distances between magnetic atomic planes under the laser-induced structure contribute to the change of magnetic interactions. Furthermore, the magnetic order can be tuned by the anti-site disorder. On the other hand, the topological order of the new laser-induced structures appears in a form of the band inversion.

In a word, it highlights the order parameters that can be changed with non-linear phonon interactions indirectly by externally pumping an infrared (IR) -active phonon mode to modulate the equilibrium of the structure into a transient state of modified orders. Besides, it provides an initiative for more detailed experimental investigations to explore light controlled orders .

We thank the referee for this comment.

Before the paper can be judged for publication, a major revision is needed. Some technical suggestions are listed below:

1. In equation 3, the spin exchange interaction J is only related to displacement μ(t). This is only true using Born-Oppenheimer approximation. When the system illuminated with a laser, there are some non-adiabatic effects. Is Born-Oppenheimer approximation appropriate in this case?

The referee is correct. Our theory works in the Born-Oppenheimer approximation. In equilibrium, the electronic gap (~80 meV, approximately 20 THz) is larger than the phonon frequencies (~5 THz for the laser-driven infrared mode and >1 THz for the breathing mode). Therefore, the Born-Oppenheimer approximation applies at the initial time.

After the laser excitation, the electronic states evolve in response to the lattice motion. In a previous work (Phys. Rev. B 104, 245135 (2021)), some of us treated the lattice and electron excitation on the same footing using a tight-binding model in a gapless system. We analyzed the adiabaticity of the laser-driven electron and phonon processes. We showed that when the laser is in resonance only with the phonons (as in 2SL MBT/MST) the electronic states preserve adiabaticity to a good approximation if the laser pulses are short enough. Such calculations are not possible for complex systems such as 2SL MBT/MST, but strongly suggest our approximations are reasonable.

However, to quantify this effect in MBT (MST is analogous), we model the material with a low-energy model and compute the single-particle electronic excitation spectrum, assuming that the laser couples with the electrons. We found that for the laser intensities required to switch the sign of the interlayer exchange interaction, the time-average (over the duration of the pulse) probability <P> of the electrons to remain in their initial state is rather high (<P>~0.7). (We explain this new calculation in detail in the new section “Single-particle electronic excitation spectrum” of the Supplemental Material.) Therefore, the approximations considered in this work are justified to a good degree.

2. In Fig. 3, $\langle Q_{R(3)} \rangle$ is ranged from 0 to 5 Å√amu. Why does $\langle Q_{R(3)} \rangle$ range from -10 to 10 Å√amu in Fig. 4?

We thank the referee for pointing this out to us. In Fig. 3, we show the $\langle Q_{R(3)} \rangle$ achieved for a given peak electric field. The first aspect is that there are no negative values for $\langle Q_{R(3)} \rangle$. This is because the phonon nonlinear coefficients (γ_3 in Eq. (1) in the main text for MBT and γ_{13}, γ_{23} in Eq. (2) in the main text for MST) have a fixed sign, which only allows $\langle Q_{R(3)} \rangle >= 0$. The maximum possible value for $\langle Q_{R(3)} \rangle \sim 5$ Å√amu is determined by the stability of the dynamical equations, which would signal material damage.

In Fig. 4 we extended this range for $\langle Q_{R(3)} \rangle > 5$ Å√amu for illustration purposes only. The negative values cannot be obtained with laser excitation, but we considered it would be useful to show that negative $\langle Q_{R(3)} \rangle$ values lead to stronger AFM coupling, in analogy with what one obtains by applying pressure to the material.

3. Is E_0 the electric field E_l in the laser or $E_l/\varepsilon(\omega)$, where $\varepsilon(\omega)$ is the dielectric function under a laser with frequency ω?

E_0 is the laser electric field. To further clarify this, we note that the polarization in MBT/MST can be written as $P = \varepsilon E_0 + Z^* Q_{IR}$, where $\varepsilon=\varepsilon(\omega)$ is the dielectric function under a laser with frequency ω (as the referee indicates). The second term of P is the ion contribution to the polarization. We assumed that the light-matter coupling is given by $E_0 * P$. Since the first term in P is independent of the phonon displacement, it does not influence the phonon dynamics.

4. How many unit cells have been used in this article? What's the meaning of N in equation $\bar{J}_{eff}=1/N\sum_{ij} J_{ij}$?

In our calculations, we used a supercell, which consists of 7 SLs of MBT (MST) and 3 SLs of vacuum simulated by empty spheres. J_{eff} represents an average exchange interaction. N means the number of interacting magnetic moments taken for the average.

To address this point, we included these details in the main text, shown in blue on page 9.

5. How did the authors define the coupling constant δJ by Floquet theory?

The coupling constant δJ arises from the Taylor expansion of the exchange interaction J_ij[Q(t)] in terms of the lattice displacement Q(t). Floquet theory provides a time-averaged picture that allows us to study the system as a function of <Q>. The value of δJ is computed fully via first-principles calculations, as shown in Fig. 4.

6. What is the time scale of the spin flipping during magnetic phase transition? Is the time scale shorter than the phonon's lifetime? i.e. Can the phase transition be observed by experiments?

We thank the referee for this question, which is similar in spirit to question 3 from the other referee. Here we reproduce the answer for the Referee's convenience.

Before the laser arrives at the sample, the electronic gap is larger than the phonon frequencies. Therefore, we can assume that the electrons follow the motion of the ions adiabatically since electrons typically have dynamics in the femtosecond regime, much shorter than the phonon lifetime (~13.3 ps). The renormalization process of the exchange interaction is thus much shorter than the phonon lifetime.

Once we obtained J_eff, as given in Fig 4, we can assume that the magnetic moments follow the Landau-Lifshitz-Gilbert equations. We estimated the time evolution of the spin aligned antiferromagnetically under the new J_eff. In our simple numerical estimate, the system becomes ferromagnetic in around 4-10 ps for J_eff ~ 0.01-0.02 meV, comparable with the phonon lifetime. However, there are several unknowns in this estimate, such as the Gilbert damping factor, the exact spin anisotropy (Phys. Rev. Lett. 124, 167204 (2020)), which can increase or decrease this estimate.

In any event, recent experiments (Nature Physics 17, 489–492 (2021)) demonstrating magnetization reversal via ultrafast phononics have shown that trains of laser pulses can be created. In principle, well-timed train pulses could sustain the distortion for longer than the phonon lifetime. In such a case, the limit would be imposed by the threshold for sample laser damage.

Therefore, we expect that the magnetic and topological transition here predicted can be observed in experiments.

7. In Fig. 5, what does band inversion mean? What's the meaning of the bands' colors?

A band inversion means a change of the character (e.g., orbital) of the bands due to the presence of strong spin-orbital interaction (SOI) in these materials. We estimate it as the difference between the spectral functions of anions (Te) and cations (Mn and Bi(Sb)). The change in the color means that the band inversion occurs. In absence of SOI, there is no band inversion and there are no topological effects.

8. Supplementary information and Appendix are absent.

We thank the referee for pointing this out. We have made sure that the Supplementary information is included in the revised version of the manuscript.

jz-2022-00070t.R2

Name: Peer Review Information for "Light-driven Topological and Magnetic Phase Transitions in Thin-layer Antiferromagnets"

Second Round of Reviewer Comments

Reviewer: 2

Comments to the Author

It is strange that I did not find a point-to-point response to the previous review comments. Instead, only a letter to the editor is shown. Usually this is not enough to judge what whether the manuscript is improved or not. Reading from the revised manuscript, it is noticed that the authors made some efforts to improve the presentation of the paper. No intention to delay its publication, the paper can now be published.

jz-2022-00070t.R3

Name: Peer Review Information for "Light-driven Topological and Magnetic Phase Transitions in Thin-layer Antiferromagnets"

Third Round of Reviewer Comments

Reviewer: 2

Comments to the Author

The authors answered the review questions and make revisions in the manuscript. I suggest acceptance of the paper.
